# A new role of growth hormone and insulin growth factor receptor type 1 in neonatal inflammatory nociception

**DOI:** 10.1097/PR9.0000000000000608

**Published:** 2017-07-13

**Authors:** Alfredo Manzano-García, Mohammed Gamal-Eltrabily

**Affiliations:** Departamento de Neurobiología del Desarrollo y Neurofisiología, Instituto de Neurobiología, Universidad Nacional Autónoma de México, Querétaro, México

**Keywords:** Nociception, Neonatal pain, Growth hormone, IGFr1

## Abstract

Growth hormone (GH) and insulin growth factor 1 (IGF1) are implicated in nociceptive processing; it has been reported that the latter participates in neonatal inflammatory nociception. In the target article, the authors propose that local inflammation evoked by carrageenan administration in mice produces a decrease in the local GH levels and an increment of IGF1 receptors type 1 expression, this produces behavioral nociception and peripheral sensitization that can be prevented by GH systemic administration pretreatment.

## Abstract 2

Neonatal inflammation produces nociception by a local decrease of growth hormone and an increment of the insulin growth factor 1-1R. Systemic growth hormone prevents the development of nociception.

**Commentary on:** Liu X, Green KJ, Ford ZK, Queme LF, Lu P, Ross JL, Lee FB, Shank AT, Hudgins RC, Jankowski MP. Growth hormone regulates the sensitization of developing peripheral nociceptors during cutaneous inflammation. PAIN 2017;158:333–46.

**Response article:** Jankowski MP. A new role of growth hormone and insulin-like growth factor receptor type 1 in neonatal inflammatory nociception: response to commentary. PAIN Reports 2017:e609.

Growth Hormone (GH) is known for its role in physical growth and neural development; many studies are being done to figure out if GH is involved in other physiological functions away from its principal role in growth. In this respect, it is believed that GH plays a role in nociceptive modulation, since patients with GH deficiency can present resting pain^1^ and GH treatment produces an analgesic response in patients with fibromyalgia.^4^

It is known that most of GH effects are mediated through insulin-like growth factor 1 (IGF1).^8^ Furthermore, IGF1 and insulin-like growth factor receptor type 1 (IGFr1) are also implicated in nociceptive modulation, especially in inflammation-induced mechanical and thermal hypersensitivity.^9^ Indeed, the IGFr1 expression is increased during neonatal inflammatory pain evoked by carrageenan (Car) administration.^7^

Presently the mechanisms implicated in neonatal pain are poorly known so understanding the singularities of neonatal pain development is essential to improve the analgesic management during this critical age. In this sense, we will discuss the study realized by Xiaohua Liu et al.^11^ in which they reported the role of the GH-IGF1-IGFr1 axis in neonatal inflammatory pain (Figure 1A) and the possible efficiency of exogenous GH as an antinociceptive treatment (Figure 1B).

**Figure 1.**
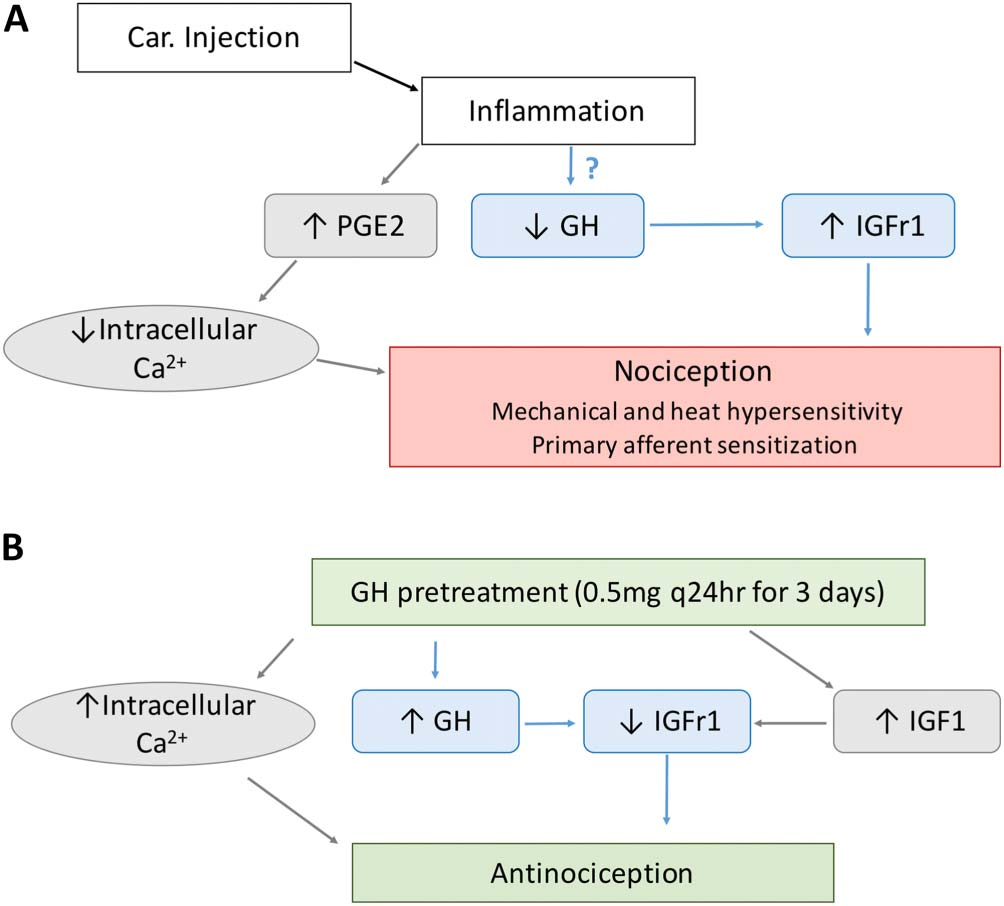
(A) Carrageenan administration in the hind paw of mice produces a nociceptive response (mechanical and heat hypersensitivity) due to upregulation of IGFr1, decreased levels of growth hormone (GH) and increased levels of PGE2 associated with decreased intracellular calcium levels. (B) GH pretreatment provokes antinociceptive response through downregulation of IGFr1, normalization of GH local levels and increased intracellular calcium. In blue, the hypothesis proposed by the target article meanwhile in grey other possible hypotheses are shown.

The experiments of this study were performed in two different cohorts of mice, 7 and 14 days after born (P-7 and P-14). This was done to evaluate if any difference was presented in 2 neonatal stages that are characterized by intense development changes in nociceptive processing. For instance, previous works described that during this transition period a switch in the function of C-fibers is presented^15^ and that the descending inhibitory systems become functional only at P-10.^5^

The authors have demonstrated using Western blot analysis that Car-induced cutaneous inflammation in the paw reduced the GH levels in a local manner one day after Car administration at both postnatal ages (P-7 and P-14). Carrageenan administration did not produce a general diminishing in GH levels, as was demonstrated by the finding that in noninflamed tissue, the GH levels remained unaltered. The decrease in GH levels was transitory and completely absent 3 days after Car administration in both groups. The causal explanation of the inflammation-induced GH local reduction was not investigated in the current study.

In both mice-cohorts, the Car-induced inflammation produced mechanical and heat hypersensitivity in behavioral pain models. Notably, the behavioral mechanical hypersensitivity and the local reduction in GH coincided temporarily, both have presented only one day after Car administration but not on 3 days post-injection. On the other hand, heat hypersensitivity was maintained for 3 days in the P-7 group and at least 7 days in the P-14 group after Car administration.

Consequently, the authors analyzed if the systemic administration of exogenous GH in 2 different treatment regimens could prevent: (1) the local decrease of GH levels and (2) the mechanical and heat hypersensitivity. Single administration of GH (0.5 mg/kg) at the same time as Car did not show efficacy in the P-14 group, and only blunted the hypersensitivity response to heat in the P-7 group. On the contrary, the application of GH 3 days before Car administration (once per day, 0.5 mg/kg) produced the normalization of local GH levels and blocked the development of hypersensitivity to mechanical and heat stimuli in both P-7 and P-14 groups.

It is interesting to point out that the GH pretreatment did not produce any effect in the basal mechanical and heat sensitivity in the P-14 group with no-induced inflammation. Nevertheless, it produced mechanical hypersensitivity and heat hyposensitivity in the related P-7 group. The exact mechanism of the contrasting GH effects in both groups was not explored during this study.

After demonstrating the behavioral antinociceptive effect of exogenous GH administration, the authors evaluated the effect of GH treatment on the primary afferents evoked activity through electrophysiological experiments. They used an interesting ex vivo preparation, consisted of isolated hairy hind paw skin, saphenous nerve, dorsal root ganglion (DRG), and spinal cord of mice. Single unit recordings were performed in the DRG neurons that innervate the area of the paw where Car was injected. Different groups of mice were assessed: (1) naive group, without any intervention, (2) P-7 group, 1 and 3 days after Car administration, and (3) P-14 group, 1 and 3 days after Car administration, and finally, the latter 2 groups after GH pretreatment (0.5 mg/kg, for 3 days). Taking into account the conduction velocity, spike morphology, and the type of stimuli that evoked the action potentials at the DRG neurons, the researchers were able to differentiate between A-fibers and C-fibers and the type of stimuli that activate them (mechanical, heat, and cold). In both groups, inflammation produced an increase in the evoked activity 1 day after Car administration, no changes were found 3 days post-injection. In the P-7 group, the increased evoked activity was mediated by A-fibers; meanwhile, in the P-14 group, this effect was mediated by C-fibers. Remarkably, the GH pretreatment in both cohorts prevented the development of the primary afferent sensitization.

Afterward, the mechanism of GH antinociceptive action was investigated. The authors found that GH did not diminish the inflammatory reaction evoked by Car, paw edema and inflammatory cytokines remained equal in treated and not treated mice. Indeed, inflammation did not produce an upregulation of GH receptors expression in DRG cells, so it was evaluated if another downstream mediator in the pathway of GH activity could be producing the GH antinociceptive effects. Jankowski et al.^7^ previously reported that Car-induced inflammation produced an increased expression of IGF1 type 1 receptor (IGFr1) in DRG cells, and it is known that this receptor is implicated in nociceptive facilitation. As a consequence, in the current work, the authors explored if GH could downregulate IGFr1. Using Western Blot analysis, it was found that the pretreatment with GH blocked the inflammation-induced upregulation of IGFr1.

Subsequently, the changes in Car-induced pronociceptive effects after IGFr1 suppression were studied. The authors used mice with nerve-specific (saphenous nerve) primary afferent knockdown of IGFr1. Then, the aforementioned behavioral and electrophysiology set of experiments were done. Remarkably, the IGFr1 knockdown mice showed a blockade in pain hypersensitivity and primary afferent sensitization in a similar manner as the GH-pretreated mice described earlier in this text.

In summary, this study suggests that GH can produce an antinociceptive effect through downregulation of IGFr1 in DRG neurons. The mechanism of the previously mentioned effect was not explored; however, the authors propose that it might be mediated by some suppressor transcription factors activated by GH (eg, ELK1) which could repress the transcription of IGFr1 gene. Though this hypothesis should be tested, we believe that a selective block of GH receptors could be considered to assure the selectivity of the GH effect. Later, selective blockade of intracellular molecular elements responsible for realizing GH actions might elucidate the exact mechanism of such an effect. Another possible mechanism of IGFr1 downregulation could be the increased level of IGF1 evoked by GH pretreatment (Figure 1A). In fact, it is known that an increment of IGF1 is a potent suppressor of the expression of IGFr1.^14^ It is worth mentioning that a nonsignificant increase in IGF1 local levels in GH-treated mice was reported in the current study as well.

Furthermore, since GH was administered systemically during this study, other antinociceptive mechanisms cannot be excluded. Considering that Car-induced pain is of inflammatory nature, prostaglandin E2 (PGE2) is one of the main mediators in this process. PGE2 was reported to present inhibitory effects on the activity of high-voltage calcium channels,^12^ as a result the intracellular levels of Ca^2+^ are decreased. An established relation between neuronal hyperexcitability and decreased intracellular Ca^2+^ is documented,^6,10^ meanwhile, boosting intracellular Ca^2+^ levels causes a decrease in the neuronal excitability.^6,13^ On a molecular level, among other intracellular mechanisms, GH promotes its effects through opening L-type Ca^2+^ channels (high-voltage channels), increasing intracellular Ca^2+^.^2,3^ Thus, we propose that another possible mechanism of the antinociceptive effect of GH could be due to the increase in intracellular Ca^2+^ that promotes a decline in the neuronal hyperexcitability induced by inflammation (Figure 1A). It could be interesting to test this alternative hypothesis.

In conclusion, Liu et al.^11^ has reported an interesting new role of the GH-IGF1-IGFr1 axis in neonatal inflammatory nociception with potential therapeutic repercussions. Since the prevention of IGFr1 upregulation is the key element to produce antinociception, it is necessary to evaluate the specific pathway involved in GH-induced IGFr1 downregulation. Additionally, it seems necessary to investigate other possible GH antinociceptive mechanisms.

## Disclosures

The authors have no conflict of interest to declare.
